# Research on Intelligent Video Detection of Small Targets Based on Deep Learning Intelligent Algorithm

**DOI:** 10.1155/2022/3843155

**Published:** 2022-07-14

**Authors:** Sucheng Kang

**Affiliations:** School of Physics and Electronic Engineering, Yancheng Teachers University, Yancheng, Jiangsu 224007, China

## Abstract

Compared with the traditional object detection algorithm, the object detection algorithm based on deep learning has stronger robustness to complex scenarios, which is the hot direction of current research. According to the process characteristics of the object detection algorithm based on deep learning, it is divided into two-stage object detection algorithm and single-stage object detection algorithm, focusing on the problems solved by some classical algorithms and their advantages and disadvantages. In view of the problem of object detection, especially small object detection, the commonly used data sets and performance evaluation indicators are summarized; the characteristics, advantages, and detection difficulties of various common data sets are compared; the challenges faced by commonly used object detection methods and small object detection are systematically summarized; the latest work of small object detection methods based on deep learning is sorted out; and the small object detection methods based on multiscale and small object detection methods based on super-resolution are introduced. At the same time, the lightweight strategy for target detection methods and the performance of some lightweight models are introduced; the characteristics, advantages, and limitations of various methods are summarized; and the future development direction of small object detection methods based on deep learning is prospected.

## 1. Introduction

The main purpose of object detection technology is to find the location of the target in the input image while determining the category attributes of the target, with the development of computer hardware and software and deep learning theory, based on the goal of deep learning.

Detection has been widely used in intelligent security, smart medical, unmanned driving, and other fields. Before the advent of deep learning, traditional object detection algorithms were mainly based on manual design features, such as Sobel edge detection features, Haar features, and Hog features, which have weak generalization ability and poor performance in complex scenarios.

Object detection is a basic computer vision task that combines the two tasks of target localization and recognition, the purpose of which is to find several targets in the complex background of the image, give each target a precise target enveloping box, and determine the category to which the target in the enveloping box belongs [1]. The popularity of deep learning has benefited from object detection technology, and at present, deep learning has been widely used in the entire field of object detection, including general object detection and domain-specific object detection. Among them, small object detection is a hot and difficult problem in the field of computer vision. Due to the limited resolution and amount of information of small targets, the task of detecting small objects has become a huge challenge in the field of computer vision at this stage. Small object detection tasks also play a very important role in civil, military, security, and other fields, such as UAV target detection of ground vehicles and pedestrians, ground target detection of remote-sensing satellite images, identification of unmanned pedestrian vehicles and traffic signs in the distance, detection of some early lesions and lumps in medical imaging, and automatic industrial inspection of small defects on positioning materials. With the gradual complexity and intelligence of computer vision systems in real life, the detection task of small targets also needs more attention.

In view of the problem of object detection, especially small object detection, this paper first summarizes the commonly used data sets, systematically summarizes the commonly used object detection methods, as well as the challenges faced by small object detection, sorts out the latest work of small object detection methods based on deep learning, briefly reviews, and finally summarizes its advantages and disadvantages and discusses its possible future development directions. The control variable method compares the detection accuracy of the same target at different learning rates to verify the limitations of the target detection method and provide the basis for the future development direction.

## 2. State of the Art

### 2.1. Small Object Detection

#### 2.1.1. Definition of Relative Scale

Define small targets from the relative ratio of targets to images. Chen et al. proposed a data set for small targets, defining small targets: in the same category, the median of the relative area of all target instances, that is, the ratio of bounding box area to image area, is between 0.08% and 0.58%. For example, in 640 × 480 pixel resolution images, targets of 16 × 16 to 42 × 42 should be considered small targets.

#### 2.1.2. Absolute Scale Definition

Small targets are defined from the absolute pixel size of the target. The most common definition comes from the MS COCO data set, which defines a small target as a target with a resolution of less than 32 × 32 pixels.

Although great progress has been made in object detection in recent years, the above methods only have a good effect on conventional target detection problems, and the extracted features have poor representation ability for small targets and less than ideal detection effects for small targets. The study of Huang et al. [2] shows that the average accuracy of small targets in detectors at this stage is about 10 times lower than that of large targets, which is not to say that convolutional neural networks are proposing. The characteristics of the performance ability are not enough, but the resolution of the small target is too low and can provide less information to the model, which is also one of the bottlenecks that currently limit the development of target detection; more and more experts and scholars will also turn their attention to the field of small target detection and carry out research, and a series of effective improvement methods have been proposed.

### 2.2. Small Object Detection Method Based on Multiscale

Multiscale prediction refers to the prediction of the class and coordinates of objects on multiple feature maps of different scales. In the early stage of the development of object detection models, representative algorithms such as YOLO and Faster RCNN only use the last layer of features of the backbone network for target detection, resulting in insufficient detection performance of small targets. For the first time in SSDs, multiscale prediction has been used to improve the detection performance of small targets. At present, the use of multiscale prediction has become a basic operation to improve the performance of small target detection.

**Figure 1:**
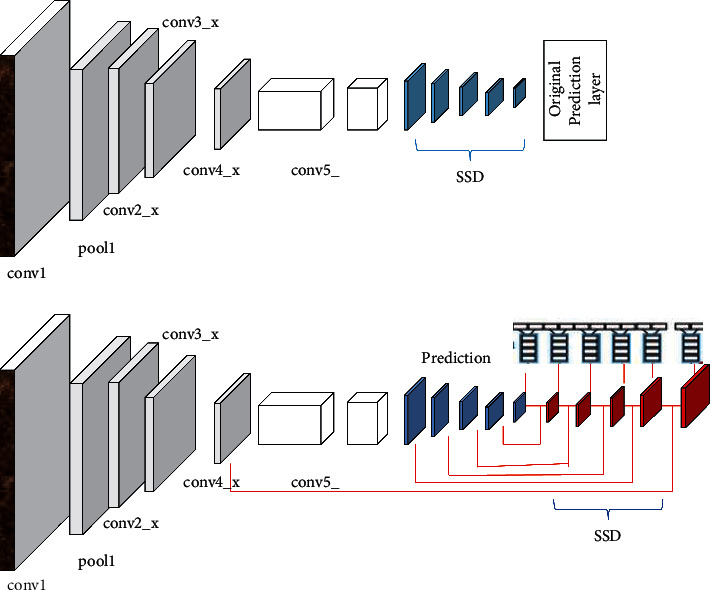
Algorithm process framework.

Most of the existing convolutional models of general object detection use the topmost features of the convolutional model for prediction, and the amount of information about small targets is less, so it is necessary to make better use of the details of the image. In a convolutional neural network, low-level features can often well represent the texture, edges, and other details of the image, while high-level features can often represent the semantic information of the image very well, but the corresponding convolutional pooling will also ignore some details. In view of this factor, Ye et al. [3] took the lead in introducing the idea of multiscale, proposed the SSD algorithm, and predicted on the feature map extracted at each scale, and the detection of small targets was better improved than that of the YOLO algorithm. Although shallow features can better represent detailed information, due to the lack of semantic information, coupled with the small target corresponding to less anchor, it cannot be fully trained, and the effect of SSD in practical applications is still not satisfactory. Fu et al. [4] made improvements to SSDs for small targets because they did not adequately correspond to anchor training as shown in Figure 1, replacing SSDs with ResNet. The VGG model, using deconvolution layers, divides the picture into smaller grids, thereby reducing the rate of missed detections. However, due to the introduction of the ResNet model by DSSD, which has more complex residual connections and lateral connections, and the addition of additional layers to the prediction module and the deconvolution module in the model, which introduces additional overhead, the DSSD algorithm is not as fast as the SSD in prediction speed algorithm. Figure 1 is the specific algorithmic process framework.

Qu et al. [5] think from the perspective of the data set, believing that the scale of the target object in the current data set is quite different, and the small target is too small compared to the picture to be detected and proposes a multiscale training method: the scale normalization of the image pyramid (SNIP), training on each scale of the pyramid, and efficiently using all the training data. Although the detection effect of small targets has been significantly improved, it is slower.

Lin et al. [6] proposed that the feature pyramid network (FPN) uses the method of collecting samples to integrate the low-level features with more detailed information and the high-level features with more semantic information. Although the efficiency of this method is low, it enhances the ability of extracting depth features of small targets, and the effect is better than general detection methods. Cao et al. combined the idea of FPN into SSD, thereby improving the detection effect of SSD algorithm on small targets, and because of its emphasis on the lightweight of the model, the parameters are slightly less, and the noise in the background information cannot be better screened out, compared with the use of feature fusion ideas. The DSSD algorithm is slightly less accurate. On the basis of FPN, Liu et al. connected the features at the bottom of the model with the features at the top level, shortened the information path between the top layer and the bottom layer, and further enhanced the connection between the feature maps of each layer.

Shrivastava et al. [7] proposed a structure similar to FPN, which realized the feature fusion of top-down in another way to improve the detection effect of small targets, and the fusion of the algorithm did not use simple weighted superposition like FPN but used convolution for fusion. The core of the algorithm lies in its top-down modulation module, the core structure of the module can be selected by itself, but because it is convoluted for feature fusion, resulting in each new module network to be gradually trained once, the training process is more cumbersome, not necessarily applicable to the actual scene. Subsequently, Ghiasi et al. and Xu et al. proposed that NAS-FPN and auto-FPN optimize the FPN algorithm, which is different. In the previous artificially designed network structure, auto-ML technology was applied to object detection so that the neural network automatically searched for the design and thus improved the efficiency of the FPN algorithm. In order to make better use of multiscale features, Guo et al. introduced a new feature pyramid structure, AugFPN, which used consistency supervision to narrow the semantic gap before feature fusion, and used residual features to reduce the convolutional pooling process. Finally, a Soft-ROI selection method is proposed to better learn the characteristics, averaged on the ResNet50 network [8].

The accuracy is improved by 2.3 percentage points, but the complexity of the model resulted in training times and frame rates using the AugFPN algorithm being inferior to that of the FPN algorithm under the same conditions, such as training each on the ResNet50 network Epoch, Faster RCNN with AugFPN takes 1.1 h, while FPN takes only 0.9 h, the frame rate is 11.1 frames and 13.4 frames, respectively. Rashwan et al. believe that the previous multiscale approach did not take into account the factors of the length and width scale, proposed the MatrixNet model, and reached 47.8% on the MS COCO data set. The average accuracy is higher than any other existing state-of-the-art single-stage object detection method, but although the method uses a matrix-based hierarchical prediction mechanism, it does not consider combining semantic information at different levels, such as high-level low-resolution and low-level high-resolution, and combining the proposed long-width scale ideas, it may be possible to go further in accuracy [9].

It can also be seen that in order to obtain better results and obtain more effective small target feature information, the multiscale detection model also changes from the initial single-layer feature to multilayer feature fusion, and the multilayer feature fusion also develops from a simple weighted superposition at the beginning to convolutional fusion and adding certain residual characteristic blocks to the model, etc. The gradual redundancy of the model can gradually improve the detection effect, but this redundancy also makes it more difficult to apply to the actual scene. Therefore, some scholars have also begun to do research on the lightweight of the model, and proposed some excellent models, such as MobileNet and ShuffleNet [10], and applied them to some existing excellent methods, such as Table 1 as shown. How to better maintain the detection accuracy while reducing the complexity of the model is also a difficult problem, while applying the lightweight model, the researchers also proposed some target detection methods that combine the lightweight strategy as shown in Table 2.

### 2.3. Small Object Detection Method Based on Super-Resolution

Because small targets occupy few pixels and have low resolution in the image, another direct way to detect small targets is to generate a high-resolution image as input to the detection model. Hu et al. used bilinear interpolation to obtain two upsampled input images to train a convolutional model, Fookes et al. use traditional super-resolution techniques to better recognize faces [11]. Although this improves the resolution of the input image which is beneficial to the detection of small targets, but also brings other problems, the super-resolution model and the detection model are trained independently of each other, the high-resolution input image generated by the super-resolution model also includes no need to detect unnecessary objects and factors, and the increase in the resolution of the input image makes the overall architecture too heavy; the training and prediction time of the model will be greatly increased, reducing the possibility of practical application. Haris et al. also proposed an architecture for end-to-end joint training of super-resolution models and detection models for this problem, but there are still a large number of images unrelated to the detection task that perform super-resolution and thus reduce the overall efficiency [12].

### 2.4. Other Methods

In addition to small object detection methods based on multiscale and super-resolution, there are some excellent methods. Takeki et al. proposed a small target detection method that combines the semantic segmentation method for the identification of small target birds in the context of the sky and takes advantage of the weak semantics of small targets to combine variants of the full convolutional network and the convolutional network and integrate the support vector machine, but it is difficult to expand only for this specific environment [13]. In the field of small object detection in remote-sensing satellite images, Ren et al. studied the RPN module of Faster RCNN and proposed the anchor corresponding to the conventional RPN module. The box scale is too large to cover small targets in the remote-sensing data set, so the RPN module corresponding to the small target scale is specially designed and combined with contextual information to improve model performance and in their self-made SORSI remote-sensing data set (containing 5 216 ship images and 706 aircraft images) achieved an average accuracy of 78.9%, but due to training samples, it still did not work well for remote-sensing targets in complex scenes and dense small optical remote-sensing targets [14]. In terms of small face detection, Zhang and others have used the difficult negative examples in OHEM to dig up ideas and dynamically at the image level and feature level. Assign the difficulty scores to the training images to determine whether they have been well detected. In this way, we can make full use of those images that have not been perfectly detected to better monitor the subsequent learning process, and achieve excellent performance on a wider range of face data sets. The subset also achieves 89.7% accuracy. Luo et al. proposed a four-branch face detection architecture that treats large, medium, and small faces separately and uses feature fusion technology to add more anchors to match small faces, which further improves the detection ability of small faces [15].

Zoph et al. said that the ability of future lightweight models to extract features is bound to be limited, starting from the data enhancement aspect is a weapon to enhance the detection effect, and assumes that when the proposed features are good enough, the use of data enhancement can get rid of the serious data-driven dependence of current algorithms. Kisantal et al. believe that there are two main reasons for the low accuracy of small target detection, one is that there are fewer pictures containing small targets in the existing public data set, and the other is that even if the pictures contain small targets, but the number of occurrences is not sufficient, the model training is not sufficient, and a means of oversampling and copying and pasting small targets are proposed for this point to enhance the data [16]. By copying the small targets in the image and pasting them to different locations in the image, the number and location diversity of the small targets in the image are increased, and the corresponding number of matching anchors will also be enhanced, thereby reducing the missed detection rate, using the Mask RCNN algorithm as a benchmark to compare the method of no data enhancement on the MS COCO data set. The detection accuracy of small targets increased by 7.1 percentage points. In addition, some scholars have also proposed some small object detection algorithms based on deep learning as shown in Table 3.

## 3. Methodology

### 3.1. Object Detection Algorithm

With the proposal of the two-stage object detection algorithm RCNN in 2014, the object detection algorithm has officially entered the era of deep learning, but the algorithm is time-consuming and cannot be practically applied. Through the in-depth understanding and improvement of the algorithm, algorithms such as SPP-Net, Fast RCNN, and Faster RCNN have been proposed one after another, and the algorithm speed has increased by hundreds of times, basically meeting the application requirements. With the increase in the application of algorithms, the shortcomings of the algorithm are also exposed, and the detection effect of small targets is not ideal is one of them; researchers through the study of feature fusion methods proposed FPN, CascadeR-CNN, M2Det, and other algorithms, which greatly improved the detection effect of small targets and the accuracy of the algorithm.

The methods of object detection are mainly divided into two categories: object detection methods based on traditional artificial features and object detection methods based on deep learning.

The concept of deep learning has its roots in artificial neural networks. The motivation for studying deep learning is to build a neural network that simulates the learning process of the human brain, and its theoretical basis is the general approximation theorem [17]. The theorem states that if enough hidden neurons are included, even if there is only one implicit layer, the expressed input-output map can adequately approximate any one continuous function defined on a unit cube. The mathematical description of the general approximation theorem is as follows:

Theorem 2.1 states that the activation function must be a nonlogarithmic, bounded, and monotonically increasing continuous function *I*_*m*_=[0,1]^*m*^, representing the unit hypercube of m-dimensional space and *C*(*I*_*m*_) representing a continuous function space on *I*_*m*_. Then, given any continuous function *f* ∈ *C*(*I*_*m*_) and *ε* > 0, there is a positive integer *n* > 0 and the real constants *a*_*i*_, *w*_*ij*_, and *b*_*i*_ (1 ≤ *i* ≤ *n*, 1 ≤ *j* ≤ *m*), which make the equation valid.

(1) $$\begin{array}{c} F\left(x_{1},x_{2},\ldots ,x_{m}\right)=\sum_{i=1}^{n}a_{i}\phi \left(\sum_{j=1}^{m}w_{ij}x_{j}+b_{i}\right). \end{array}$$

For all *X*_1_, *X*_2_,… in the input space, *X*_*m*_, satisfies relational:

(2) $$\begin{array}{c} \left|F\left(x_{1},x_{2},\ldots ,x_{m}\right)-f\left(x_{1},x_{2},\ldots ,x_{m}\right)\right|<\varepsilon . \end{array}$$

### 3.2. Gradient Descent Method

In the process of function *f* constantly approximating the F function, it is necessary to use the gradient descent method to determine the new direction of each iteration so that the objective function to be optimized is gradually reduced. The gradient descent method is the basic method for calculating the minimum values of continuously differentiable functions without constraints. Let *f* (*x*) be continuously distinct around *x*_*k*_, such that *x* = *x*_*k*_ + *αd*, where *d* is the unit direction (‖*d*‖=1). If *g*_*k*_=∇*f*(*x*_*k*_) ≠ 0 so, you get the Taylor unfolding formula.

(3) $$\begin{array}{c} f\left(x\right)=f\left(k\right)+\left(\nabla f\left(x_{k}\right)\right)\left(x-x_{k}\right)+ο\left(\left\|x-x_{k}\right\|\right), \end{array}$$

(4) $$\begin{array}{c} f\left(x_{k}+\alpha \;d\right)=f\left(x_{k}\right)+\alpha g_{k}^{T}d+ο\left(\alpha \right),\;\alpha >0. \end{array}$$

The angle between *d* and −*g*_*k*_Θ satisfies the relation

(5) $$\begin{array}{c} g_{k}^{T}d=-g_{k}\cos\;\theta . \end{array}$$

When *θ*=0, *g*_*k*_^*T*^*d* is the minimum, and in this case, *d*=−*g*_*k*_. Thus, the negative gradient direction −*g*_*k*_ is the direction in which the function *f* (*x*>  decreases fastest around *x*_*k*_.

If *f*(*x*)=0.5*x*^*T*^*Gx*, where *G* is a symmetric positive definite matrix of *n* × *n*, then the convergence rate of the gradient descent algorithm is linear, and the resulting point column {*x*_*k*_} is all *k* which satisfies the relational.

(6) $$\begin{array}{c} \frac{f\left(x_{k+1}\right)-f\left(x^{n}\right)}{f\left(x_{k}\right)-f\left(x^{n}\right)}\leq \frac{{\left(\lambda_{1}-\lambda_{n}\right)}^{2}}{{\left(\lambda_{1}+\lambda_{n}\right)}^{2}}, \end{array}$$

(7) $$\begin{array}{c} \frac{\left\|x_{k+1}-x^{n}\right\|}{\left\|x_{k}-x^{n}\right\|}\leq \sqrt{\frac{\lambda_{n}}{\lambda_{1}}}\frac{{\left(\lambda_{1}-\lambda_{n}\right)}^{2}}{{\left(\lambda_{1}+\lambda_{n}\right)}^{2}}, \end{array}$$

where the minimum values *x*^*n*^ are *f*(*x*) and the maximum *λ*_1_ and *λ*_2_ are minimum eigenvalues of *G*, respectively.

All the training data are required during each iteration of the gradient descent method, and each iteration takes a long time when the data volume is large, resulting in the whole process converging very slowly, so there are various deformations, including SGD (Stochastic Gradient Descent), Momentum SGD AdaGrad (Adaptive Gradient), RMSProp (Root Mean Square prop), Adam (Adaptive Moment estimation), and AdaDelta (Adaptive delta) [18].

SGD updates the current weight (*∂L*/*∂W*)*∗αw* using a gradient, and its iteration relationship is as follows:

(8) $$\begin{array}{c} w_{t+1}=w_{t}-\alpha \frac{\partial L}{\partial W_{t}}, \end{array}$$

where *L* represents the error between the actual output of the sample *x*_l_ and the desired output.

Momentum SGD updates the weights using the exponential moving average *V* of the historical and current gradients, with its iterative relationship:

(9) $$\begin{array}{c} w_{t+1}=w_{t}-\alpha V_{t}, \end{array}$$

(10) $$\begin{array}{c} V_{t}=\beta V_{t-1}+\left(1-\beta \right)\frac{\partial L}{\partial w_{t}}, \end{array}$$

where *V* is initialized to a zero matrix and *β* = 0.9.

AdaGrad updates the weights using the learning rate *a* divided by the square root of *S*, the gradient part being the same as SGD, but constructing an iterative relationship.

(11) $$\begin{array}{c} w_{t+1}=w_{t}-\frac{\alpha }{\sqrt{S_{t}+\varepsilon }}\cdot \frac{\partial L}{\partial w_{t}}, \end{array}$$

(12) $$\begin{array}{c} S_{t}=S_{t-1}+{\left(\frac{\partial L}{\partial w_{t}}\right)}^{2}, \end{array}$$

where *S* is the accumulation of historical and current gradient squares, usually initialized to a zero matrix, and *ε* is the parameter that ensures that the denominator is not 0 *a*=0.01, *ε*=10^−7^.

RMSProp is an improvement over AdaGrad, using an exponential moving average of gradients instead of cumulative square gradients and updating the weights, iterative relational:

(13) $$\begin{array}{c} w_{t+1}=w_{t}-\frac{\alpha }{\sqrt{S_{t}+\varepsilon }}\cdot \frac{\partial L}{\partial w_{t}}, \end{array}$$

(14) $$\begin{array}{c} S_{t}=\beta S_{t-1}+\left(1-\beta \right){\left(\frac{\partial L}{\partial w_{t}}\right)}^{2}, \end{array}$$

where *S* is initialized to a zero matrix *a*=0.01,  *ε*=10^−7^, *β* = 0.9.

Adam's iterative relationship is a combination of Momentum and RMSProp. The gradient section, like the Momentum SGD, uses the exponential moving average of the gradient instead of the current gradient update weights.

The learning section is learned using the RMSProp learning rate divided by the exponential moving average of the gradient.

(15) $$\begin{array}{c} w_{t+1}=w_{t}-\frac{\alpha }{\sqrt{{\hat{S}}_{t}+\varepsilon }}\cdot {\hat{V}}_{t}, \end{array}$$

(16) $$\begin{array}{c} {\hat{V}}_{t}=\frac{V_{t}}{1-\beta_{1}^{t}}, \end{array}$$

(17) $$\begin{array}{c} {\hat{S}}_{t}=\frac{S_{t}}{1-\beta_{2}^{t}}, \end{array}$$

(18) $$\begin{array}{c} V_{t}=\beta_{1}V_{t-1}+\left(1-\beta_{1}\right)\frac{\partial L}{\partial w_{t}}, \end{array}$$

(19) $$\begin{array}{c} S_{t}=\beta_{2}S_{t-1}+\left(1-\beta_{2}\right){\left(\frac{\partial L}{\partial w_{t}}\right)}^{2}, \end{array}$$

where *v* and *s* are initialized to a zero matrix *β*_1_=0.9, *β*_2_=0.999,  *a*=0.01, *ε*=10^−6^*a*=0.01, *ε*=10^−6^.

### 3.3. Traditional Object Detection Algorithms

Early object detection algorithms were mostly built on manual features. First, the areas that may have a target are found on the original image of the input, then the feature extraction is performed on each region, and the classifier model is sent to the classifier model for judgment, and finally, the area that the classifier model considers to be the target is filtered and other postprocessing operations are used to obtain the results. Due to the lack of efficient image representations at the time, people had no choice but to design complex feature representations and use various acceleration techniques to exhaust limited computing resources [19].

### 3.4. VJ Detector

Nineteen years ago, Viola and Jones designed an efficient face detector that was dozens of times faster than other detectors at the time, a milestone in the development of face detection and even computer vision, which was named Viola–Jones (VJ) in honor of this work. VJ Detector adopts the detection method of sliding window, uses Haar features to describe each window, and introduces an integral graph to accelerate the extraction of Haar features so that the computational complexity of each window is independent of window size combined. The AdaBoost algorithm selects features and introduces cascading ideas to reduce the amount of background window computation, increase the target computation of faces, improve accuracy, and reduce the scale of computation.

### 3.5. HOG Features

The directional gradient histogram (HOG) feature was first proposed as a local feature by Daal et al. for pedestrian detection problems. As the name suggests, HOG features accumulate to form a histogram by calculating gradient values in different directions in an area of the picture as a feature of this area [20]. HOG features can better extract the local detail information of the image, in the case of geometric deformation of the image, optical distortion, etc., and have a good feature invariance; for many years, HOG features have been the basis of many object detectors and various types of computer vision systems.

### 3.6. Variable Part Model (DPM)

Deformable part model is a deformable part model or DPM model for short. This model is very intuitive, modeling the target object into a combination of several parts. For example, it sees humans as a combination of head/body/hands/legs. Although DPM seems to be very simple, how to translate this intuition into a mathematical model is very challenging (SVM, convex optimization, coordinate descent, gradient descent). It was not until 2005 that this model was proposed because it involved a lot of optimization/training methods. After being proposed, DPM became a big hit, but after the deep learning technology caught fire, DPM seemed to lose its light. If you visualize the features of each layer of deep learning, you can see that deep learning also seems to be learning the characteristics of these components (human head/hand/foot), but deep learning is relatively simple to train. DPM, on the other hand, adds more heuristic rules and becomes more complicated. In view of the poor performance of the HOG feature processing occlusion problem, in 2008, Felzenszwalb et al. proposed the DPM algorithm and then jointly with Girshick et al. made various improvements. He excelled at the time and won the VOC Challenge in 2007, 2008, and 2009. The DPM algorithm adopts a “divide and conquer” idea, which can treat the training detection process as the learning of various parts of the object and the collection of individual parts detection and improve the HOG features. Blocks in the HOG feature are removed, and only the cells are retained.

## 4. Result Analysis and Discussion

### 4.1. Accuracy and Recall

Prior to evaluation, each predicted value will be compared to a confidence value, which is a positive sample if it is greater than that value, or an inverse sample otherwise. As a binary classification problem, there are four possibilities for the algorithm to detect the results, as shown in Table 4, the prediction is a positive sample, and the actual positive sample is called a real example; The prediction is a positive sample, and the actual is an inverse sample, called a false positive case. Predicted to be an inverse sample, the actual is an inverse sample, called a true counterexample. The prediction is an inverse sample, and the actual positive sample is a false counterexample.

During the inspection process, the usage formula (20) calculates the accuracy of the inspection, and the application formula (21) calculates the recall rate of the test.

(20) $$\begin{array}{c} \text{Precision}=\frac{TP}{TP+FP}, \end{array}$$

(21) $$\begin{array}{c} \text{Recall}=\frac{TP}{TP+FN}. \end{array}$$

### 4.2. PR Curve and ROC Curve

**Figure 2:**
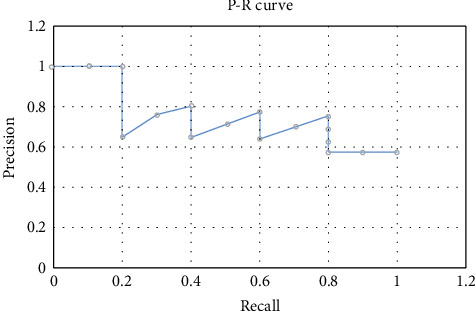
PR curve operation diagram.

ROC curves were first used to analyze radar signals in World War II to detect enemy forces. The cause is the Pearl Harbor incident; because it is more useful, it is slowly used in some applications such as psychology and medicine, and in machine learning, data mining, and other fields to judge the quality of classification and test results. PR curves and ROC curves are commonly used evaluation methods in categorical imbalance problems. For the same model, both PR and ROC curves can illustrate certain problems, and the two have a certain correlation. The PR curve is used to describe the relationship between accuracy and recall. According to the PR curve to judge the detection effect of an algorithm, there are three judgment methods: (1) according to the size of the closed area of the curve and the coordinate axis to judge the detection effect of the algorithm, the larger the closed area, the better the detection effect of the algorithm. (2) Use the balance point to judge the detection effect of the algorithm, that is, the recall rate is equal to the accuracy rate, and the larger the value of the balance point, the better the detection effect of the algorithm. (3) The detection effect of the algorithm is judged by using the F1 value; as shown in the formula, the larger the F1 value, the better the detection effect of the algorithm. The graph of the PR curve for detecting the target is shown in Figure 2.

**Figure 3:**
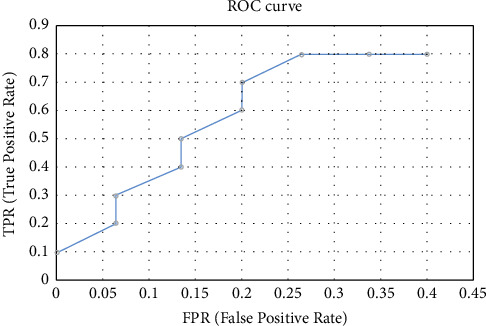
ROC curve operation diagram.

When running the target detection algorithm, the ROC curve mainly calculates the TPR and FPR. The FPR is actually the recall, which is consistent with the recall in the PR curve. The graph of the ROC curve for detecting the target is shown in Figure 3.

(22) $$\begin{array}{c} F_{1}=\frac{2*P*R}{P+R}, \end{array}$$

where *P* is the accuracy and *R* is the recall.

Since the setting of network training hyperparameters is also a key factor that directly affects the detection accuracy, the influence of different learning rates, batch sizes, and gradient descent methods on the detection accuracy is analyzed experimentally to obtain better hyperparameters.

#### 4.2.1. Learning Rate

**Figure 4:**
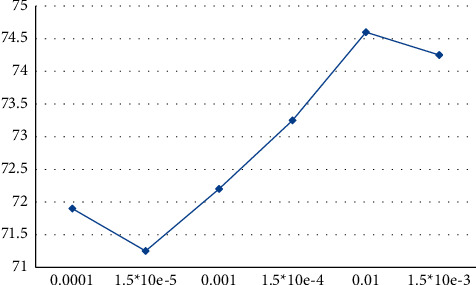
Learning rate-target detection accuracy.

Figure 4 shows the accuracy of the corresponding target detection at different learning rates.

By comparing the detection accuracy of the target at different learning rates, it can be obtained that the detection accuracy is the highest when the learning rate is 0.01.

#### 4.2.2. The Size of the Data Batch

Table 5 shows the accuracy of target detection corresponding to different batches.

By comparing the detection accuracy of the target under different batches, it can be obtained that the impact of the batch size on the detection results is not obvious, but when the batch is less than 10, the training is difficult to converge.

#### 4.2.3. Gradient Descent Mode

**Figure 5:**
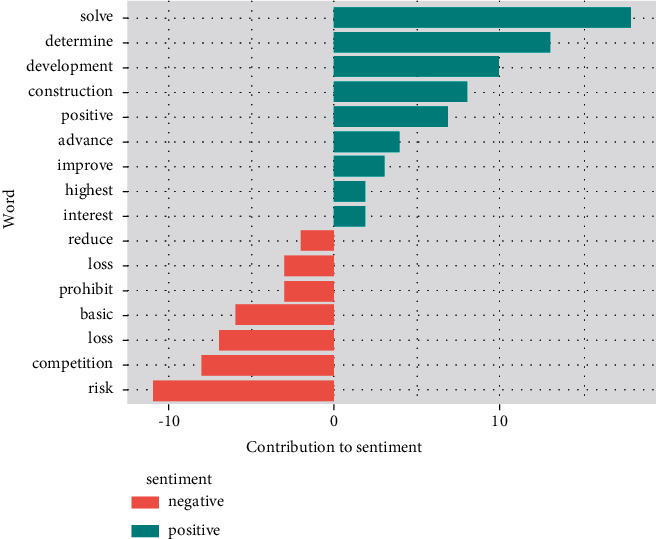
The convergence result of the iteration.

Here are the six gradient descent methods for SGD, Momentum SGD, AdaGrad, RMSProp, Adam, and AdaDelta. The convergence effect of 1000 times was compared, and the convergence result of the iteration is shown in Figure 5.

Through the above comparative experiments, it can be found that among the six iteration methods, the convergence is faster and more stable under the AdaDelta gradient descent method, while the gradient descent method affects the convergence speed of the algorithm, thereby affecting the number of iterations of the algorithm.

Based on the above analysis, during the experiment, the learning rate was set to 0.001, the batch was set to 25, and AdaDelta was used as the gradient descent method for this article.

Experiments and analysis show that the above improvement strategy can maintain the accuracy of ordinary object detection while greatly improving the accuracy of small object detection of SSD algorithm so as to meet the requirements of conventional target detection tasks. With the improvement of shooting technology and the change in the shooting environment, the requirements for small object detection have gradually increased. The further research directions of this paper are reflected in: (1) the method of augmenting the small target data, exploring a better way of augmenting the small target data, and determining its relationship with the detection accuracy; (2) aiming at the proposed contextual feature fusion structure, the detection speed of the network is improved by simplifying the number of convolutional layers of the underlying network; and (3) continue to optimize the improved NMS method so that the network can adaptively select a better reward parameter.

## 5. Conclusion

This paper systematically elaborates the research progress in the field of object detection in recent years, including traditional object detection methods and deep learning-based object detection methods, and reviews and analyzes the popular related data sets. Focusing on the more difficult small object detection problems in the field of object detection, he analyzed some improved algorithms at home and abroad in the small object detection problem in recent years, hoping to bring new research ideas to researchers in related fields. Although the existing small object detection algorithm has achieved some results, but the accuracy is still very low, with the gradual complexity of the computer vision system deployed in real life, the accuracy requirements of small object detection will gradually improve, through the summary and analysis of the above technology, and the following points are proposed.

1. Small target detection combined with traditional methods
2. Introduce attention mechanisms
3. Build a more complete small object detection data set
4. Lightweight of the mold type to improve the real time, accuracy, and robustness of the detection system
5. In the process of model training, focus on training for small goals
6. Research on small object detection method based on anchor-free

In view of the above problems, this paper makes some prospects for the future development of deep learning-based object detection algorithms. For the problem of low detection accuracy of small targets, the anchor-based algorithm is mainly due to the fact that the anchors set up cannot match the small targets well, resulting in the inability to extract the characteristics of the small targets well. The D2Det algorithm improves the matching effect of small targets and anchor by introducing dense local regression, thereby improving the detection effect of small targets, and in the future, it can also improve the detection accuracy of small targets on this basis. For the problem of unbalanced positive and negative samples during training, OHEM, Focal Loss, GHM, and other algorithms balance the loss feedback brought by positive and negative samples to the network by manually suppressing negative sample losses, and in the future, they can more reasonably balance positive and negative sample losses through online adaptive methods to further improve the algorithm effect. Some application areas due to confidentiality are difficult to obtain, resulting in less training sample size; you can simulate the scene to increase the sample size, you can also do some enhancement and expansion of samples through the GAN series algorithm, and you can also further study the relevant methods of small sample learning and improve the training effect of small sample set.

## Figures and Tables

**Table 1:** Brief introduction and comparison of lightweight networks.

Model name	Reference volume/10^6^	Basic introduction	Classification effect on ImageNet/%
MobileNet v1	4.20	Lightweight network model that can be used for mobile, using deep separable convolution instead of normal convolution; the number of parameters and the amount of computation are greatly reduced, but the straight-cylinder structure is not sufficient for feature learning	70.6
MobileNet v2	3.40	The hourglass residual structure is introduced to enhance the gradient propagation and reduce the computation, and the ReLU function is removed from the last layer to preserve the feature diversity	72.0
MobileNet v3	5.40	The model structure is improved by using the network structure search algorithm, while the SE module is introduced to strengthen the network learning ability by combining with the channel attention mechanism, and the h-swish activation function is proposed to improve the accuracy	75.2
ShuffleNet v1	1.90	The point-by-point group convolution is used to reduce the computational complexity of 1 × 1 convolution, and the channel transformation method is proposed to induce the flow of information in the same channel of different features, but the difference between the number of input and output channels is still too large to affect the efficiency	67.8
ShuffleNet v2	2.30	Abandon group convolution and introduce channel splitting operation to reduce the number of network branches to obtain faster detection speed with certain accuracy	69.4
ShuffleNet	1.25	Replace the 3 × 3 convolution with 1 × 1 convolution, reduce the number of convolution channels, and postpone the sampling operation to significantly reduce the number of parameters and the amount of computation so as to maximize the speed increase in exchange for the reduction in accuracy	57.5

**Table 2:** Target detection methods combined with lightweighting strategies.

Method name	Year	Basic introduction	Performance
CSPNet	2019	Starting from the perspective of network architecture, we use cross-stage feature fusion to optimize the repetitive gradient information in the network to achieve lightweighting	In the same environment, the computation is reduced by nearly 30% and the accuracy is improved by 2% compared to yolov3
YOLO Nano	2019	Design PEP macro-architecture by human-machine collaborative design strategy, combined with fully connected attention module for embedded environment to significantly reduce the amount of computation, but only in embedded environment	Achieve an average accuracy of 69.1% on the VOC2007 data set and a model size of only 4 MB
ThunderNet	2019	Based on ShuffieNet v2, compressing RPN module, proposing context enhancement module, using 1 × 1 convolutional compression channel to achieve feature fusion effect while reducing computational cost, and introducing spatial attention mechanism to optimize feature distribution to reduce computational effort	Obtain 19.1% AP on the COCO data set, which is similar to the accuracy of SSD using MobileNet, but nearly 5 times faster and significantly cheaper to compute
RefineDeLite	2020	A new backbone network Res2NetLite is proposed for the detection task, ensuring the same number of input and output channels, focusing on optimizing the loss function and training strategy	The AP value of 26.8% is achieved on the COCO data set, and it can reach 29.6% with its proposed training strategy, which is the best lightweight network at present

**Table 3:** Other deep learning-based methods for small target detection.

Order number	Title	Main content	Year
1	Small object detection with random decision forests	A small target detection algorithm based on antimachine straight forest is proposed for small-shaped UAV target detection task, achieving 98.8% and 98.7% on single-day and multiday subsets of UIUC data sales	2017
2	Small object detection using context and attention	The multiscale features are concatenated and the additional features of different spreading are used as contexts, and a target detection algorithm combining the note checking force mechanism is also proposed, and the detection effect of both methods on small targets is higher than SSD	2019
3	Bond curated: Online semantic similarity small object detection in crowded scenes	Introducing a pairwise constraint referring to semantic eclipse and using the contextual information of candidate objects to improve the detection performance of tiny objects	2019
4	Lightweight small target detection algorithm based on improved SSD	Adding transposed volumetric station structure to SSD algorithm to improve small target detection	2018
5	An Atrous filter design to enhance the detection capability of small targets in SSD	Based on the SSD algorithm, the special evidence fusion of three or four convolutional layers is performed and undergoes a strong splitting of the empty volume to improve the accuracy and robustness of detection	2019
6	Real-time small target detection method based on improved PVANet	To improve the candidate pivot selection method to better locate small targets based on PVANet for real-time small target detection problem	2020
7	Multiscale Faster RCNN detection algorithm for small targets	Improve the structure of Faster RCNN network, while using the high- and low-level features of the network and use the crawler crawl data to increase or decrease the training data set	2019
8	A small object detection solution by using super-resolution recovery	Segmentation of original aerial images and semi-super resolution for enhancement using GAN	2019
9	TBC-Net: a real-time detector for infrared small target detection using semantic constraint	Proposing a lightweight slow network TBC-Ne for out-of-river small target detection, and adding high-level semantic constraint information of images in the training process to solve the problem of uneven streets of small target samples	2019
10	Multiscale convolutional feature fusion for SSD target detection algorithm	Regional amplification extraction of model low-level features and fusion with high-level features to improve small target detection	2019
11	Improvement of YOLO3 in aerial target detection	Reduce some of the convolution operations and introduce the jump connection on the basis of the original model to ensure the real-time performance	2020
12	Deep learning image target detection combined with note checking force mechanism	Introducing an attention mechanism module in the two-order strand detection model associated with subregion features and competitive height ratio properties	2019
13	Visual small object detection in SSD based on feature union	Fusing feature information from deep and found layers and adjusting the prior to small day marker size for better small target detection	2020

**Table 4:** Classification table of forecasts.

Real situation/predicted results	Positive sample	Antisample
Positive sample	TP (real example)	FN (false counterexample)
Antisample	FP (false-positive example)	TN (true counterexample)

**Table 5:** The accuracy of target detection corresponding to different batches.

Bulk	30	25	20	Bulk	30	25	20
Aircraft	83.6	82.1	80.1	Dogs	87	82.7	83.1
Bicycles	85.1	82.7	83.1	Ma	83.1	84.1	84.9
Bird	73.5	72.5	75.9	Motorcycle	81.3	79	82.6
Boat	60.6	66.5	64.3	People	80.5	76	78.5
Bottle	46.2	47.2	51.3	Potted plants	45.9	45.8	48.2
Buses	82.4	81.8	81.2	Sheep	75.2	74.7	73.4
Small cars	81.5	83.8	81.9	Sofa	78.5	75.9	75.3
Cat	89.2	84.3	86.4	Trains	83.9	85.2	82.5
Chairs	55	58.4	54.4	Monitors	72.5	73.7	74.8
Dairy cattle	79	79.4	80.3	mAP	74.89	74.6	74.8
Dining table	73.8	76.1	73.8	—	—	—	—

## Data Availability

The labeled data set used to support the findings of this study is available from the author upon request.
